# The HOG Pathway Is Critical for the Colonization of the Mouse Gastrointestinal Tract by *Candida albicans*

**DOI:** 10.1371/journal.pone.0087128

**Published:** 2014-01-27

**Authors:** Daniel Prieto, Elvira Román, Inês Correia, Jesus Pla

**Affiliations:** Departamento de Microbiología II, Facultad de Farmacia, Universidad Complutense de Madrid, Plaza de Ramón y Cajal s/n, Madrid, Spain; Instituto de Salud Carlos III, Spain

## Abstract

The opportunistic pathogen *Candida albicans* is a frequent inhabitant of the human gastrointestinal tract where it usually behaves as a harmless commensal. In this particular niche, it needs to adapt to the different micro environments that challenge its survival within the host. In order to determine those factors involved in gut adaptation, we have used a gastrointestinal model of colonization in mouse to trace the behaviour of fungal cells. We have developed a genetic labelling system based on the complementary spectral properties of the fluorescent proteins GFP and a new *C. albicans* codon-adapted RFP (dTOM2) that allow a precise quantification of the fungal population in the gut via standard *in vitro* cultures or flow cytometry. This methodology has allowed us to determine the role of the three MAP kinase pathways of *C. albicans* (mediated by the MAPK Mkc1, Cek1 or Hog1) in mouse gut colonization via competitive assays with MAPK pathway mutants and their isogenic wild type strain. This approach reveals the signalling through HOG pathway as a critical factor influencing the establishment of *C. albicans* in the mouse gut. Less pronounced effects for *mkc1* or *cek1* mutants were found, only evident after 2–3 weeks of colonization. We have also seen that *hog1* mutants is defective in adhesion to the gut mucosa and sensitive to bile salts. Finally, we have developed a genetic strategy for the *in vivo* excision (tetracycline-dependent) of any specific gene during the course of colonization in this particular niche, allowing the analysis of its role during gut colonization.

## Introduction

The fungal pathogen *C. albicans* is a common inhabitant of certain body locations, such as the gastrointestinal and vaginal tracts, where it frequently behaves as a harmless commensal; however, an alteration of host defense mechanisms may lead to a pathogenic behavior of the fungus. Under certain circumstances, this process may involve the translocation of the fungus to the bloodstream from these reservoirs, reaching essential organs and causing severe diseases. Despite the increasing importance of non-*Candida* and non-*albicans* species [1], this fungus is the 4^th^ leading cause of nosocomial infections, representing a primary health problem in several countries that is partially aggravated by the relatively limited antifungal therapy available [2], [3]. Several approaches have been developed to understand its mechanisms of virulence, although the concept of virulence may not be easy to be defined for “pathobionts” [4]–[6]. Development of genetic tools for this fungus (see [7] for a recent review) has allowed, nevertheless, the identification of several “virulence” factors, mainly using the mouse intravenous model to test their role during a systemic infection. They include adhesion molecules that facilitate the interaction with the host cells, metabolic or nutritional traits that optimize growth *in vivo* and several other factors that facilitate invasion. The ability to switch between different morphologies (called polymorphism) is also an important feature that influences pathogenicity as it facilitates tissue penetration and escape from phagocytes [8], [9]. Factors affecting *C. albicans* commensal colonization of different mucosal surfaces may represent an alternative promising approach in the control of candidiasis [6], [10]. In fact, high fungal gastrointestinal levels may be an important predisposing factor towards acquired *Candida* infections [11], which come mainly from endogenous origin [12]. Although some non-vertebrate models have been developed in the last years to analyse fungal virulence [13], the use of mammals becomes more relevant when considering the similarity between the immunological system and the routes of infection of rodents and humans. Commensalism models [14] are acquiring increasing relevance compared to the standard systemic models. These models make use of either neonatal or adult immunocompromised mice or animals partially depleted in the gastrointestinal microbiota by the use of a broad spectrum antibiotic therapy [15]. Combined genetics and transcriptional analyses have revealed the role of certain metabolic traits [16], transcription factors [17], [18] and phenotypic switch-related genes [19] in the adaptation to the gastrointestinal niche.

Mitogen-activated protein kinase (MAPK) pathways represent one of the main mechanisms of adaptation to environmental changes in *C. albicans*. These signaling pathways are present in all eukaryotic cells and sense changes on different situations like osmotic, oxidative or nitrosative stress, pH alterations, temperature, nutrient starvation or when cell wall damage is induced by certain drugs (i.e. Congo Red, azoles, and cell wall synthesis inhibitors). Upon perception of an external stress, via a membrane receptor, they generate a signal by sequential phosphorylation that finally leads to an adaptive response via specific transcription factors. Previous work from our group -and other laboratories- has shown that MAPK mediated signaling pathways play an important role in virulence (see [20], [21] for recent reviews). This concept fits well with the existence of specific conditions on certain body locations during infection [22], [23]; in fact, oxidative stress is a common challenge for any pathogen as immune cells like macrophages and neutrophils continuously sample microbial cells and destroy them [24]. In *C. albicans*, three main MAPK pathways have been characterized. The cell integrity pathway involves the Mkc1 MAPK [25] and participates in cell wall construction and host interaction [26]–[28]. The HOG pathway relies on the Hog1 MAP kinase, enabling adaptation to both osmotic and oxidative stress [29]–[32]; this route, however, also participates in the biogenesis of the cell wall [29], [33]–[35]. Finally, the Cek1 kinase participates in morphogenesis, invasion and cell wall biogenesis [36], [37]. Despite this apparently pleiotropic nature, each pathway responds to specific stimuli and generates a rather specific response, although it is clear that several cross talk mechanisms exist among them [38], [39]. Genetic analysis has revealed that mutants defective in each pathway show a reduced virulence in the standard mouse systemic model of infection and some of them also show reduced virulence on certain alternative models of virulence [20].

We are interested in studying the role of these MAP kinases routes in a model of sustained gut colonization [40] and we have, therefore, extended this model to specific conditions where long-term high-level *C. albicans* sustained colonization is achieved; in our model, two different strains can be assessed together and easily detected by both culture-dependent and independent methods. We demonstrate, using this methodology, that *mkc1* and *cek1* mutants are defective in long term sustained colonization while the absence of a functional HOG pathway results in a complete failure to establish and to maintain stables fungal loads in the gut, indicating that the signaling through this route is a critical adaptive mechanism to colonize the mouse gastrointestinal tract.

## Materials and Methods

### Ethics Statement

All the animal experiments performed in this work were carried out in strict accordance with the regulations in the “Real Decreto 1201/2005, BOE 252” for the Care and Use of Laboratory Animals of the “Ministerio de la Presidencia”, Spain. The protocol was approved by the Animal Experimentation Committee of the University Complutense of Madrid (Permit Number: BIO2012-31839-1). None of the treatments resulted in noticeable disease in the animals as determined by external or internal (*post mortem*) examination, still all efforts were made to minimize suffering. Mice euthanasia was performed by CO_2_ inhalation following standard protocols (AVMA Guidelines for the Euthanasia of Animals: 2013 Edition). The number of animals used in the experimentation was minimized for ethical reasons.

### Strains and growth conditions

All *C. albicans* strains used in this work, listed in Table S1, derive from the SC5314 clinical isolated [41]. The parental strain CAF2 is referred here as wild type control [42]. Strains defective in *MKC1*, *CEK1* or *PBS2* genes were described elsewhere [25], [43], [44]. Conditional and mutant *HOG1* strains are described in the next section.

Yeast strains were routinely short-term stored at 4°C and grown at 37°C in YPD medium (2% glucose, 2% peptone, 1% yeast extract) or SD medium (2% glucose, 0.5% ammonium sulphate, 0.17% yeast nitrogen base) plus amino acids and chloramphenicol (10 µg/mL). When necessary, to regulate the TET expression system, 20 µg/mL doxycycline (Sigma) was added to the medium. Nourseothricin was added (200 µg/mL) for selection of *C. albicans* transformants. Drop tests were performed by spotting 5 μL drops of 10-fold serial dilutions of stationary grown cells onto YPD plates supplemented with bile salts (Sigma) or SDS (Duchefa Biochemie) at the indicated concentrations. Plates were incubated for 48 h at 37°C and scanned for figure assembly.

Plasmids were propagated in *Escherichia coli* strain DH5α. *E. coli* strains were grown in LB medium either with ampicillin (100 µg/mL) or chloramphenicol (20 µg/mL) according to the plasmid marker.

### Molecular biology procedures and plasmid constructions

pNIM1R, which allows a repressible tetracycline-dependent regulation (TET-OFF) in *C. albicans*, was kindly provided by Dr. L.E. Cowen (unpublished data). Adapted GFP [45] was obtained from pNIM1_MoGFP_carboxi_ca_myc [46] by digestion with *Sal*I-*Bgl*II restriction enzymes. This piece of DNA was ligated with a *Sal*I-*Bgl*II fragment from pNIM1R to generate pNIM1R-GFP.

A red fluorescent protein (RFP) suitable for expression in this fungus (called here *C. albicans* dTOM2, Figure S1) was synthesized by codon optimization of the DsRed-derived RFP dTomato gene [47] based on codon usage of four highly expressed *C. albicans* genes (*HWP1*, *ENO1*, *MRPS9* and *ACT1*) as template (GenScript, USA). The dTOM2 gene was cloned into pNIM1R by replacing the GFP fragment after digestion with *Sal*I and *Not*I restriction enzymes, therefore generating a tetracycline-repressible RFP plasmid. Both pNIM1R-GFP and pNIM1R-dTOM2 were used to generate fluorescent labeled strains. Products of plasmid digestion with *Kpn*I and *Ksp*I, that directs integration at the *ADH1* region of wild type or mutant strains of *C. albicans*, were transformed by electroporation and transformants were selected as nourseothricin resistant clones. Strains' genotype was confirmed by PCR analysis.

To obtain the plasmid pNIM1-FLP-URA3 (flippase gene expressed under TET-ON regulation), plasmid pNIM1 [48] was digested with *Sal*I and *Ava*I restriction enzymes and ligated with a FLP-URA3 *Ava*I fragment. The latter was amplified from pSFL213 [49] using the primers o-FLPURAup (AGGCTCGAGATGTCACAATTTGATATATTATGTAAAAC) and o-FLPURAlw (TCCCCCGAGTTATAATTGGCCAGTCTTTTTCAAAT).

The *hog1* mutant used in this work was obtained following the standard *URA3*-blaster method [42] in a CAI4 strain [42] instead of our previous published *hog1* mutant (in RM1000 background) [50] to avoid side effects due to chromosome 5 aneuploidy [51]. The HOG1cR strain was generated as follows. First, a *hog1* mutant was transformed with a DNA construct in which the *HOG1* gene was flanked with FRT (flippase recognition target) recombination sites that directed this piece of DNA to the *ARD1* locus [52], thus obtaining the strain HOG1fR. Then, the *FLP* gene expressed under TET-ON system in pNIM1-URA3-FLP was introduced by homologous recombination at the *ADH1* region using enzymes *Kpn*I and *Ksp*I, as previously described.

### *In vivo* procedures

Female mice C57BL/6 were obtained from Harlan Laboratories Inc. (Italy) and used within an age of 7 to 10 weeks-old. Mice housing and other non-invasive procedures took place in the animal facility from the Medical School of the Universidad Complutense de Madrid.

Colonization assays to test the establishment of *C. albicans* commensal in the gut began with a four days antibiotic pretreatment followed by a single gavage of 10^7^ yeast cells in 100 µL of sterile PBS. Unless otherwise indicated, the standard antibiotic treatment in sterile drinking water consists of 2 mg/mL Streptomycin (Sigma), 1 mg/mL Bacitracin (Sigma) and 0.1 mg/mL Gentamicin (Sigma) as described elsewhere [40]. Fresh stool samples were periodically collected (every 2–4 days) from each individual and mechanically homogenized in PBS to quantify the fungal population by Colony Forming Units (CFUs) determination on SD-agar plates. For flow cytometry analysis, the homogenized stool samples were immediately filtered through a 40 µm-cell strainer nylon filter (BD) prior to its analysis in a Guava EasyCyte cytometer. Samples were fixed using a 1% formaldehyde solution for 30 minutes at 4°C. To determine *C. albicans* population in different gut locations, mice were sacrificed and intestinal tracts were aseptically exscinded. Samples from cecum, small and large intestine were homogenized in sterile PBS and 10-fold serial dilutions were cultured to determine *C. albicans* CFUs.

### FACS and microscopy analysis

Epifluorescence microscopy images were obtained from an Eclipse TE2000-U inverted microscope (Nikon) coupled with an Orca C4742-95-12 ER Charge Coupled Device camera (Hamamatsu). Capture and image processing were performed with AquaCosmos Imaging System 1.3 software. Guava EasyCyte cytometer and InCyte software (Millipore) were used for flow cytometry qualitative and quantitative analysis of fluorescence labeled *C. albicans* strains.

### *Ex vivo* adhesion assay to intestine mucosa

For the *ex vivo* adhesion assay, a 1 cm-piece of the large intestine was obtained from a recent euthanatized mice. This piece was longitudinally opened, carefully washed with sterile PBS and placed in a 4 mm-diameter methacrylate chamber filled with RPMI media pre-warmed at 37°C. *C. albicans* cells from an overnight YPD culture were suspended in RPMI serum-free media at a concentration of 2.5×10^7^ cells/mL. The lumen side from the colonic tissue was inoculated with 10^6^ yeast cells an incubated for 150 minutes at 37°C. After this period, the piece of intestine was carefully washed with sterile PBS twice and mechanically disaggregated. *C. albicans* cells recovered from the latter fraction were considered as adhered and further analysed by CFU standard determination.

### Statistical analysis

Statistical differences among two groups were calculated using Student's two-tailed unpaired *t*-test. Data are expressed as each replicate and median (colonization assay) or mean ± standard deviation (adhesion assay).

## Results

### Dual fluorescence genetic labelling allows *C. albicans* discrimination in mouse fecal samples

**Figure 1:**
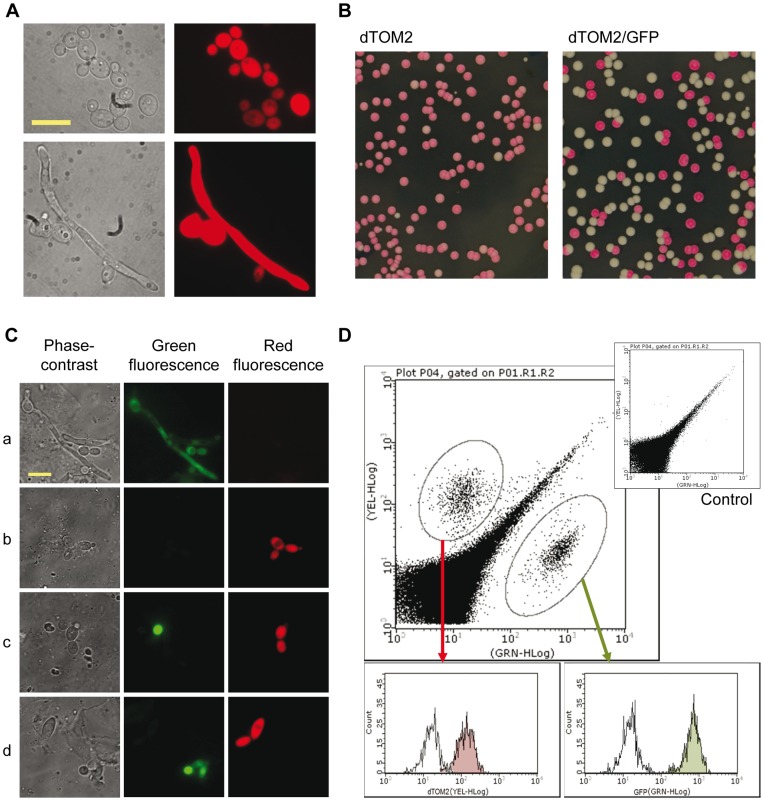
Detection of *C. albicans* GFP and dTOM2 *in vitro* and in fecal samples. A) Phase contrast (left) and fluorescence (right) images showing the expression of dTOM2 in exponentially growing cultures of CAF2-dTOM2 strain in YPD (upper panel) or YPD supplemented with 10% serum (lower panel). Bar indicates 10 µm. B) Appearance of colonies from a pure CAF2-dTOM2 (left, dTOM2) or a mixed CAF2-dTOM2/CAF2-GFP culture (right, dTOM2/GFP) in SD plates after 2 days of growth at 37°C. C) Detection of fungal cells in four different fecal samples by fluorescence microscopy. Left, phase contrast; center, green channel and right, red channel. Bar indicates 10 µm. D) Flow cytometry profile of a fecal sample from a mouse simultaneously colonized with two different labelled fluorescent *C. albicans* strains. Main panel shows the dot-plot with green (GFP) and red (dTOM2) populations while the panels below show the histograms of the correspondent gated regions (circled), shown as a coloured filled histogram, with a histogram from an unlabelled control (empty histogram). As additional control, a dot-plot of a fecal sample from a non-colonized mouse is shown in the upper part of the figure (control).

In order to determine the behavior of mutant strains of *C. albicans* in the gut, we devised a competitive assay that would allow a precise determination of fitness within the gastrointestinal tract. In this scheme, two strains are labeled with different and distinguishable fluorescent proteins so the evolution of the population can be determined by analysis of the intestinal content or stool samples. Since its adaptation to *C. albicans*, green fluorescent protein (GFP) has been broadly used in genetics [53], allowing precise detection of gene expression in cells by both fluorescent microscopy and flow cytometry [45], [54]. We have used in this work the GFP allele from Dr. J. Morschhäuser [45], called MoGFP or simply GFP. In order to use a complementary fluorescent marker, we adapted the DNA sequence of the RFP dTomato [47] (see Material and Methods). The expression of this gene from the tetracycline-dependent integrative plasmid pNIM1R allowed detection of cells expressing the RFP *in vitro*, in the absence of doxycycline on liquid media in both yeast and hyphal forms (Figure 1A). No signal was detectable when doxycycline was present in the medium (not shown) in accordance with the tight regulation of this system in yeast [55]. Expression in *C. albicans* was also detectable in SD plates as pink-reddish colored colonies after two days of growth and these colonies were clearly distinguishable from GFP-expressing ones on a plate (Figure 1B). When samples were taken from mice colonized with a mixed population of both CAF2-GFP and CAF2-dTOM2, microscopic examination of fresh stool samples also allowed the identification of GFP and dTOM2 expressing cells (Figure 1C). We also analysed fecal samples using a protocol for flow cytometry in order to achieve an instantaneous culture-independent analysis (see Material and Methods). FACS analysis of stool samples showed the appearance of a positive population for each signal, either in the green (GFP) or yellow (RFP) channel, which were not observed in similar samples from non-colonized control mice (Figure 1D). Mean fluorescent intensities under these conditions were 126 for dTOM2 (yellow channel) and 667 for GFP (green channel), enough to distinguish from non-labeled controls (means of 16 and 14 in yellow and green channels, respectively) in dot-plot or histograms (Figure 1D). In non-colonized mice, common events, that would correspond to microbiota population with varying degrees of autofluorescence, were detected (Figure 1D, upper small dot-plot). This analysis could be prolonged in animals colonized for up to 60 days with similar results (data not shown) indicating its independence on the timing of gut colonization. We also found a good correlation of the quantification of the population between both culture dependent (standard CFU counting) and independent (flow cytometry) measurements (Figure S2A). These results indicate that genetic labelling allows a precise *in vitro* and *ex vivo* quantification and analysis of populations of *C. albicans* strains colonizing the mouse gut.

### Gut adapted *C. albicans* cells are not able to compete with unaltered endogenous microbiota

The ability of *C. albicans* to colonize murine gastrointestinal tract has been studied by some authors, revealing the need of diminishing the bacterial microbiota to allow the establishment of this fungus within the gut [14], [40]. In some of these models, stable long-term *C. albicans* gut colonization is achieved if oral antibiotic therapy is established before a single gavage of this fungus in mice and maintained along the experiment. We have studied the role of antibiotic therapy both in the establishment and in the maintenance of *C. albicans* colonization.

**Figure 2:**
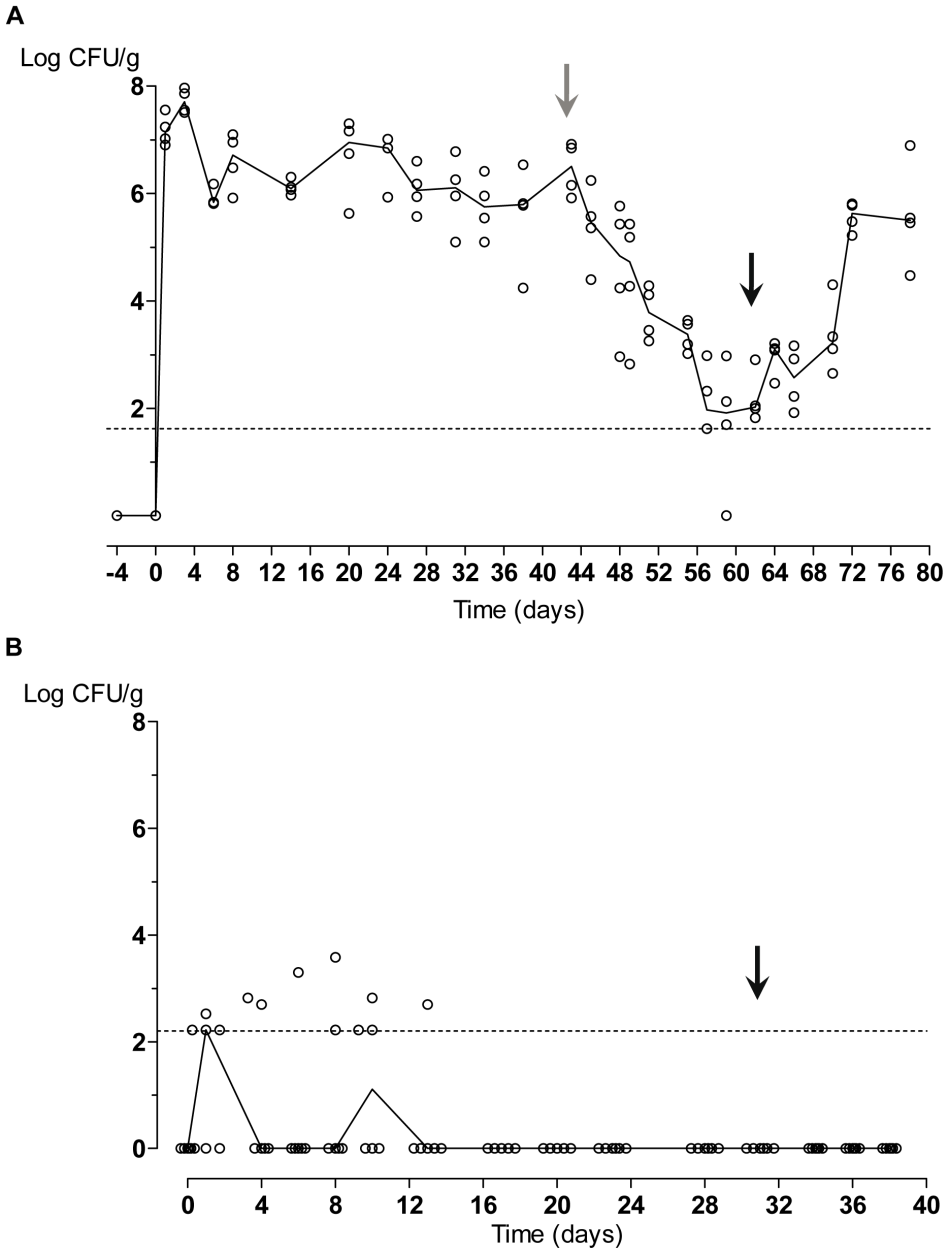
Effect of antibiotic therapy upon *C. albicans* gut colonization in mice. *C. albicans* colonization assays. CFUs from each individual are represented as open circles, while the line reflects the tendency of the median. Horizontal dashed line refers to the detection limit. A) Oral antibiotic therapy (streptomycin, bacitracin and gentamicin) was given to mice (n = 4) from 4 days before a gavage of 10^7^ *C. albicans* wild type cells (day 0) and maintained until day 43 when it was removed (grey arrow) and restored again on day 62 (black arrow). B) Mice (n = 6) were gavaged with 10^7^ *C. albicans* wild type cells and analysis of CFUs in stools was followed as a function of time. Oral antibiotic therapy was administered from day 31 (black arrow).

First, we have determined the relevance of *C. albicans* adaptation during long term colonization. For this, we kept this fungus colonizing mice for 6 weeks (43 days) to allow a long term adaptation to the commensal state in the presence of antibiotic therapy that was then removed (Figure 2A, grey arrow). Suppression of oral antibiotic therapy led to an immediate loss of fungal population in stool samples, which were barely detectable after additional 15 days. The kinetics of fungal loss indicates an almost lineal drop of 0.306 logarithmic units per day in the fungus content per gram of feces (r^2^ = 0.98). This event was reversible if the fungus was not completely absent from the animals since oral antibiotic reestablishment (black arrow) allowed recovering original high levels colonization without additional inoculation of *C. albicans* cells (Figure 2A). The increase in colonization was estimated to be 0.273 logarithmic units per day (r^2^ = 0.73).

In a separate experiment, we confirmed the fate of ingested *C. albicans* cells in non-antibiotic treated mice by counting CFUs in stools shortly after ingestion. As seen in Figure 2B, doses up to 10^7^ cells did not end up in sustained colonization; rather, a transient passage through the gastrointestinal tract was observed. At initial days a few individuals (1 to 3 out of 6) showed a value of log_10_CFUs around 2.2, slightly above the limit of detection in these analyses. These data indicate that, albeit transient, a low level of *C. albicans* (<10^3^ CFU/g) does indeed exist. Although this is an erratic short-term colonization and no fungus was detectable later on, we tried to discard the existence of cryptic low latent colonization of *C. albicans* in mice. Treatment with oral antibiotics at day 31 did not end up with an increase in *C. albicans* population to detectable levels, even though this event was observed in the previous experiment when antibiotic therapy was reestablished (Figure 2A). These data collectively indicate that normal mice microbiota is an effective barrier for the establishment of *C. albicans* within the mice gut even when the fungus is already adapted to the gut niche.

### Mutants defective in the HOG pathway are unable to establish gut colonization

Previous studies have revealed a role for the MAP kinases pathways in virulence in *C. albicans* in models of systemic infection. We addressed here the importance of *C. albicans* MAPKs in its ability to establish colonization within the mouse gut applying the model described. For this kind of experiments, a mixed population of a wild type and mutant strain expressing either dTOM2 or GFP were administered by gavage to antibiotic treated mice. The population from stools was subsequently analysed on SD plates, discriminating each strain population by the different colony pigmentation. We first confirmed *in vitro* that expression of either GFP or dTOM2 did not significantly alter the behaviour of *C. albicans* as the duplication time in exponentially growing phase was not altered (67 min for all strains,Figure S2B), either in the presence or absence of 20 µg/mL doxycycline which regulates the expression of fluorescent proteins. In addition, a 50% mixed population of wild type cells (CAF2-dTOM2 and CAF2-GFP) did not result in a substantial reduction of the percentage of either GFP or dTOM2 expressing *C. albicans* cells in SD cultures *in vitro* (Figure S2C) even after a significant number of duplication times (>50 generation times) during 11 sequential exponential growths. This mixed population also behaved neutral in an *in vivo* colonization experiment in mice (Figure S2D).

**Figure 3:**
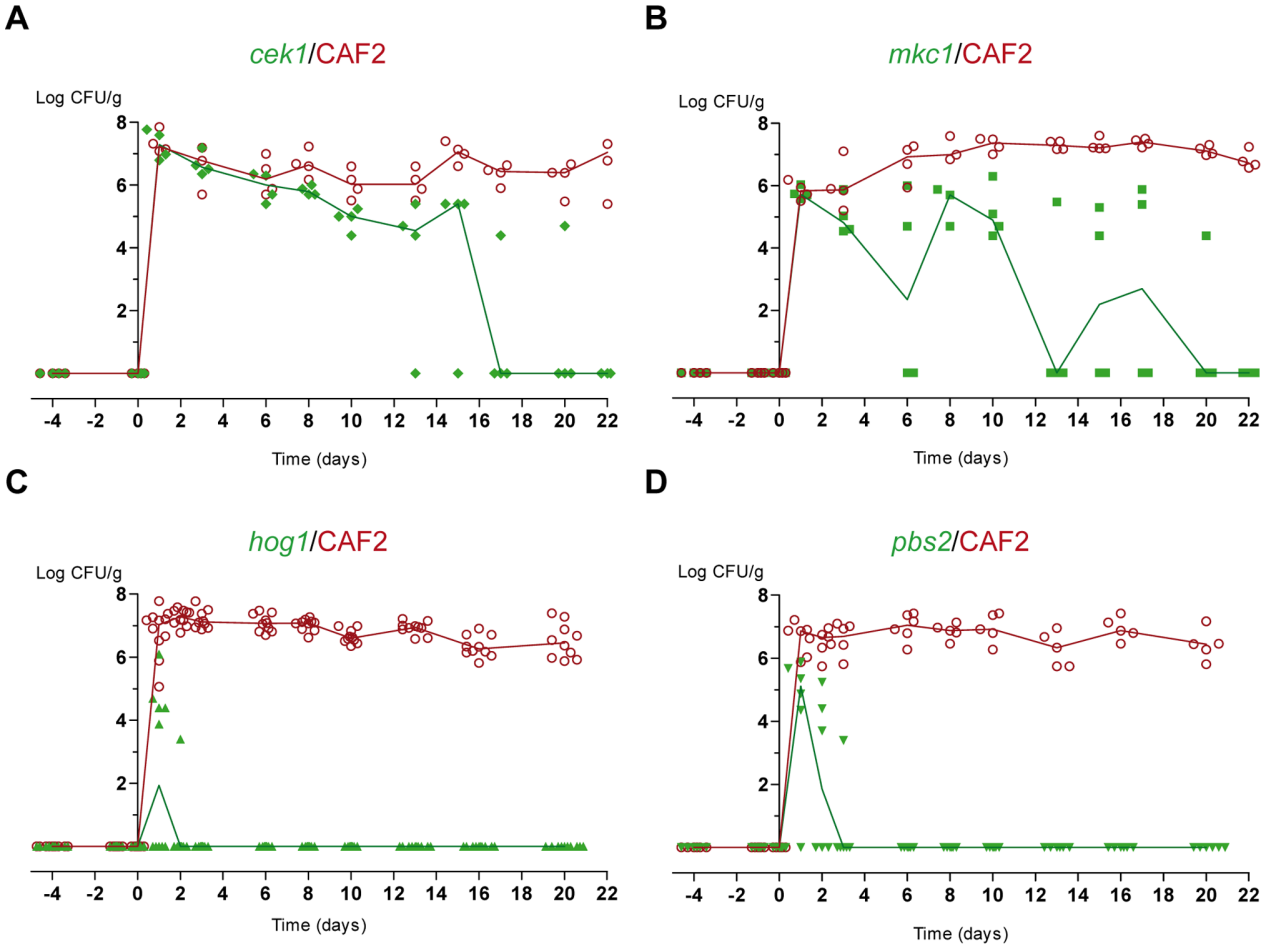
Fitness of MAPK mutants in *C. albicans* in gut colonization. Competition colonization assays with MAPK mutants. CFUs from each individual are represented as open red circles (CAF2-dTOM2) or green symbols (mutant-GFP). Colored lines reflect the tendency of the median of the respective strain. Oral antibiotic therapy (streptomycin, bacitracin and gentamicin) was given to mice (n = 4–6) from 4 days before a gavage of 10^7^ cells in a 1 1 mixture of CAF2-dTOM2 and mutant-GFP (day 0). CFUs were counted and associated with each strain based on their pattern of fluorescent protein expression on solid SD plates. Mutants assessed were A) *cek1* (diamonds), B) *mkc1* (squares), C) *hog1* (triangles) and D) *pbs2* (inverted triangles).

We therefore tested the MAPK mutants in this model. As shown in Figure 3, the relevance of each MAPK in gut establishment is clearly different. The behaviour of *cek1* and *mkc1* mutants was similar, reaching levels comparable to the wild type strain at early time points (day 1) but gradually disappearing after 2-3 weeks (Figures 3A and B). In stark contrast, *hog1* mutants were unable to establish within the gut when co-inhabiting with the parenteral strain (Figure 3C). In fact, we were only able to detect very low levels of *hog1* mutant cells (in the range of 10^3^–10^4^ CFU/g) and only at the earliest time points (days 1 and 2). This kinetic is consistent with simple intestinal transit but not establishment as occurs with non-antibiotic treated mice. To assess whether this phenotype is characteristic of HOG pathway activation, we tested the behaviour of *pbs2* mutants (which are defective in the Hog1-specific activator MAPKK [44]). While there were certain differences (*pbs2* population peaked higher at 10^5^ CFUs/g than those of *hog1* mutants) the maximum CFU detection also took place at day 1 and disappeared in about 3 days (Figure 3D).

**Figure 4:**
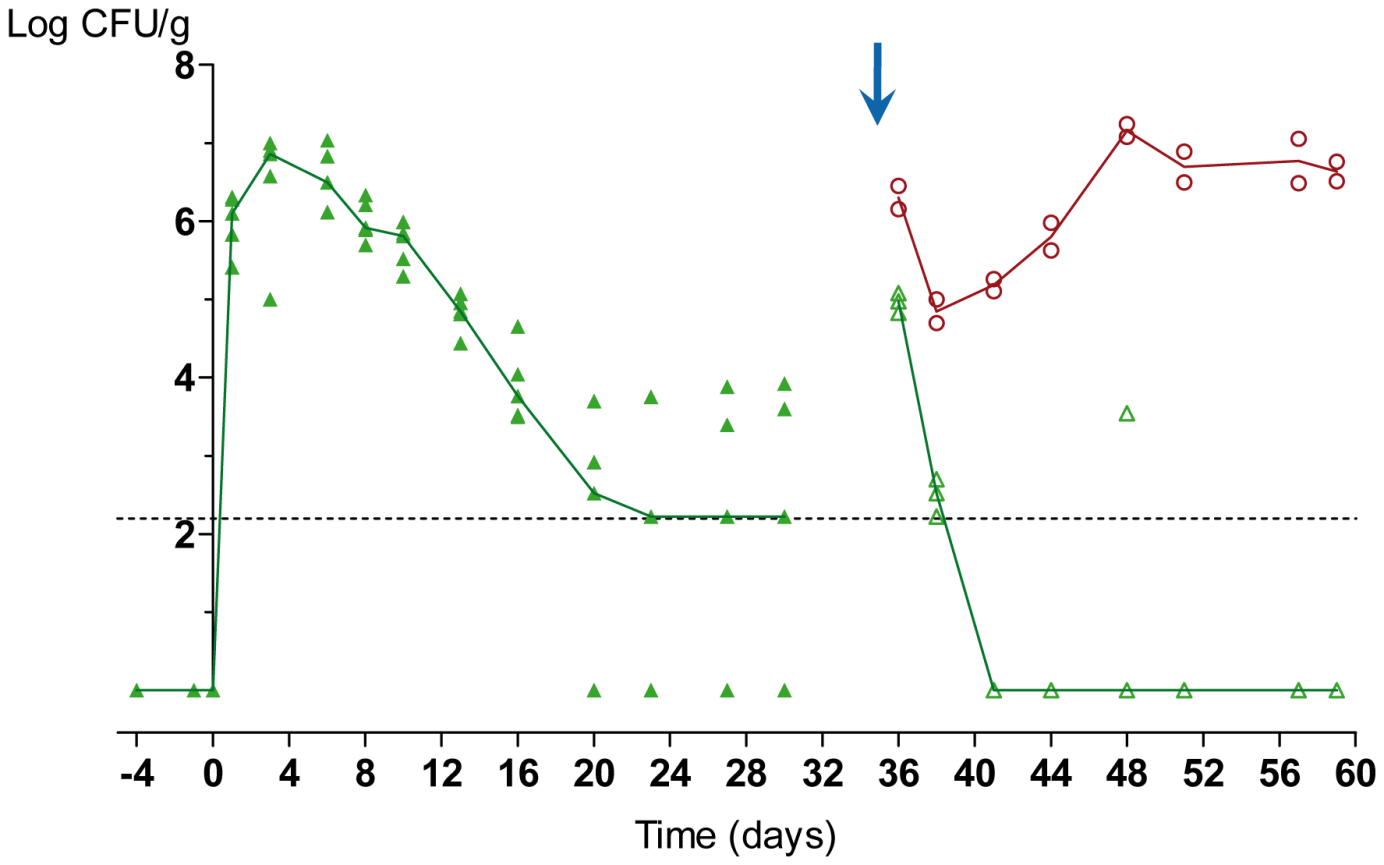
Gut colonization of the *hog1* mutant. *C. albicans hog1* mutant colonization assay. CFUs from each individual are represented as green triangles (*hog1*-GFP) or red circles (CAF2-dTOM2), open symbols refer to the analysis after the re-inoculation of the respective strains on day 35 (blue arrow). Colored lines reflect the tendency of the median of the respective strain. Oral antibiotic therapy (streptomycin, bacitracin and gentamicin) was given to mice (n = 5) from 4 days before a gavage of 10^7^ cells of *hog1*-GFP (day 0). *hog1*-GFP and CAF2-dTOM2 strains were inoculated (10^7^ cells) on day 35 after splitting the group of mice into two separate cages (blue arrow). CFUs were counted and associated with each strain based on their pattern of fluorescent protein expression on solid SD plates.

We wanted to confirm that colonization impairment also takes place in the absence of wild type cells. When a GFP-labeled *hog1* strain was inoculated to colonize mouse gut under standard conditions, it reached a colonization level of 10^6^ CFUs/g constant over a period of 6–8 days. Afterwards, it showed a gradual decrease in population detected from individuals stools until day 20 (reduction of 0.324 logarithmic units per day in the fungus content per gram of feces, r^2^ = 0.96) followed by high variable values, most of them around or below our detection limit (Figure 4).

Since in this experiment we lacked an internal control, we wondered if this failure to colonize was due to the *hog1* phenotype or an artifact caused by a physiological change in the animals or the selection of an intrinsically competitive microbiota. This does not seem to be the case, since inoculation of two separated mice from this group with a wild type CAF2-dTOM2 strain still allowed a high level (standard) colonization (Figure 4, open circles), while after re-inoculation the rest mice of group with a fresh *hog1*-GFP strain (obtained from *in vitro* culture), did not allow detection of fungal cells in stool samples in the following days (Figure 4, open triangles).

### *HOG1* expression is necessary for long term colonization of *C. albicans* to the mouse gut

Due to the defects present in the *hog1* mutant that impair initiating gut colonization, it is difficult to assess whether Hog1 has an actual role in the maintenance of *C. albicans* in the gut. We devised a complementary strategy for checking the behaviour of a fungal population in which excision of *HOG1* gene is induced *in vivo* in the gut. To obtain a *HOG1* conditional mutant, we transformed a *hog1* mutant strain with a construction in which the *HOG1* gene and the *SAT1* (nourseothricin marker) were flanked by flippase FRT repeats [52] and ectopic expression of the FLP recombinase gene was dependent on a TET-ON expression system (see Material and Methods and Figure S3B). This strain, HOGcR, reverts *in vitro* the main *hog1* phenotypes such as osmo-sensitivity and oxidative stress sensitivity unless FLP is induced by doxycycline addition to the medium (data not shown). We previously checked that the TET-ON system efficiently expresses GFP *in vivo* (Figure S3A), so we expected that administration of tetracycline (in this case autoclaved chlortetracycline or aCT, a tetracycline derivate that lacks most of the antibiotic activity) would trigger an efficient *in vivo* FLP-mediated *HOG1* excision. This process occurred very efficiently *in vitro* on cells growing in YPD-20 µg/mL aCT with more than 99% of cells showing osmo-sensitivity (*hog1* phenotype) after 48 hours, (Figure S3C).

**Figure 5:**
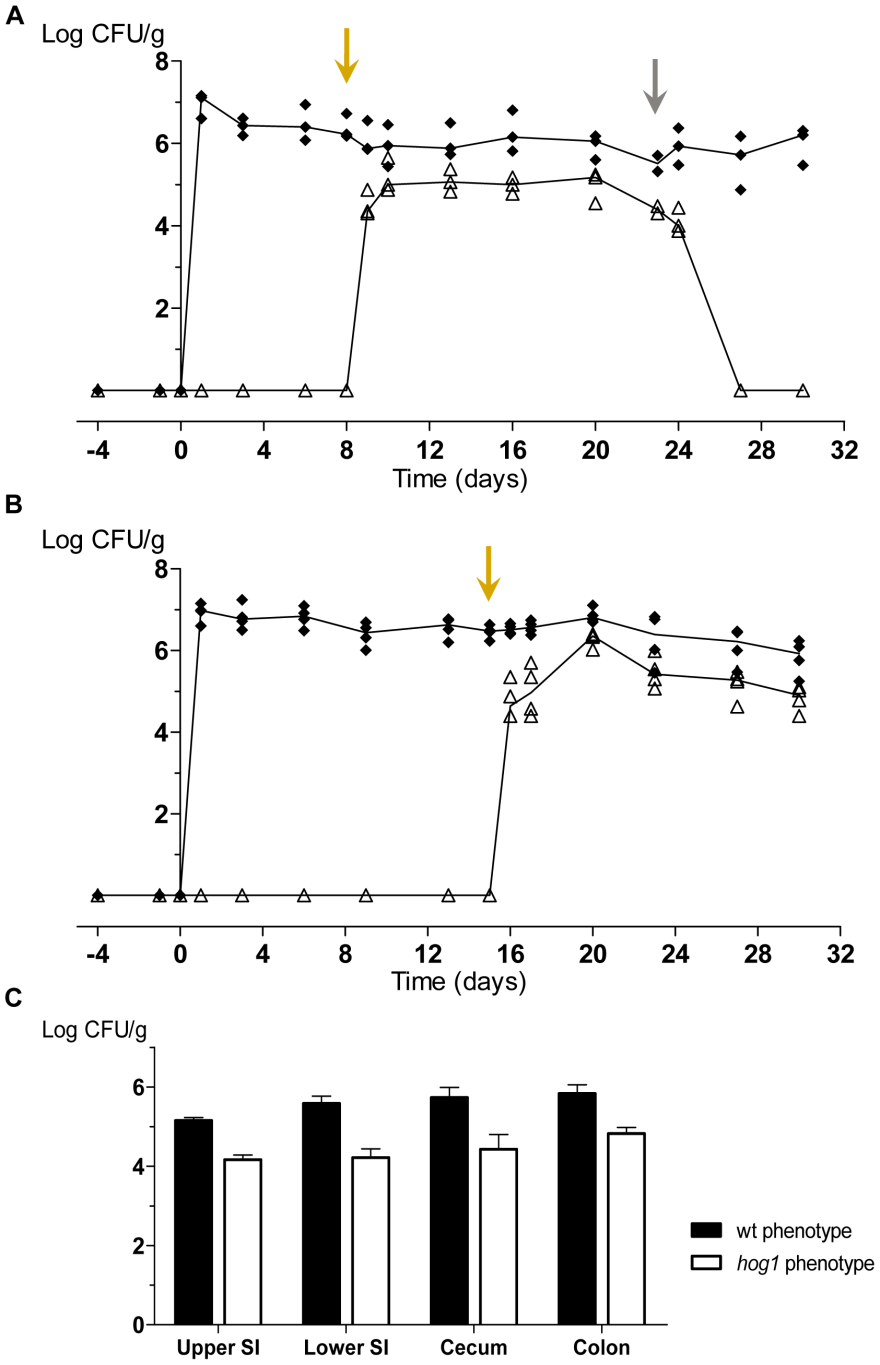
*In vivo* excision of *HOG1* gene during colonization. A-B) *C. albicans* HOGcR strain colonization assay. CFUs from each individual are represented as closed diamonds (wt-phenotype) or open triangles (*hog1*-phenotype). Lines reflect the tendency of the median of the respective population. Oral antibiotic therapy (streptomycin, bacitracin and gentamicin) was given to mice (n = 3–4) from 4 days before a gavage of 10^7^ cells of HOGcR strain. Autoclaved chlortetracycline (1 mg/mL aCT) was added to oral treatment to induce the expression of *FLP* gene (yellow arrow) or removed in order to stop it (grey arrow). CFUs were counted and associated with each population based on their pattern of osmo-sensitivity on 1.5 M sorbitol solid YPD plates and confirmed by nourseothricin sensitivity. C) Intestines from mice in Figure 5B were dissected on day 30 and each location was homogenized and analysed in fungal content. CFUs were counted and associated with each population based on their pattern of osmo-sensitivity on 1.5 M sorbitol solid YPD plates and confirmed by nourseothricin sensitivity. Black colums refers to CFUs population showing wt-phenotype, while white columns refers to *hog1* phenotype-population. SI  =  small intestine.

Upon oral colonization, HOGcR showed standard levels of colonization, reaching values up to 10^6^–10^7^ cells/g in stool samples, with all cells (>99,9%) showing a wild type phenotype (osmo-resistance). However, from the first day after instauration of aCT therapy (yellow arrow), we observed the appearance of CFUs with a *hog1* phenotype (osmo-sensitive) from stools, even though a osmo-resistant wild type population remained almost constant (Figures 5A and B). Osmo-sensitive population levels were around 10^5^ CFUs/g, still 10 fold less than wild type cells (around 10^6^ CFUs/g) and were constant over the entire period where the aCT therapy was maintained (Figures 5A and B), but it rapidly disappeared once aCT was removed from oral treatment (Figure 5A, grey arrow). Analysis postmortem of intestinal samples revealed that in all gut locations (small and large intestine and cecum), the proportion among *HOG1*-excised and non-excised populations remained nearly the same (Figure 5C). However, it is important to notice that osmo-resistant colonies recovered from these mice were not refractory to FLP expression, since excision of *HOG1* could be efficiently achieved *in vitro* by aCT (data no shown). A explanation for this behaviour would be that FLP mediated excision is not completely efficient *in vivo* and that mutant cells do not outcompete wild type cells, further demonstrating an important role for Hog1 also once high fungal loads have been already established.

### *Hog1* role in mucosa adhesion and *in vitro* resistance to the gut surroundings

**Figure 6:**
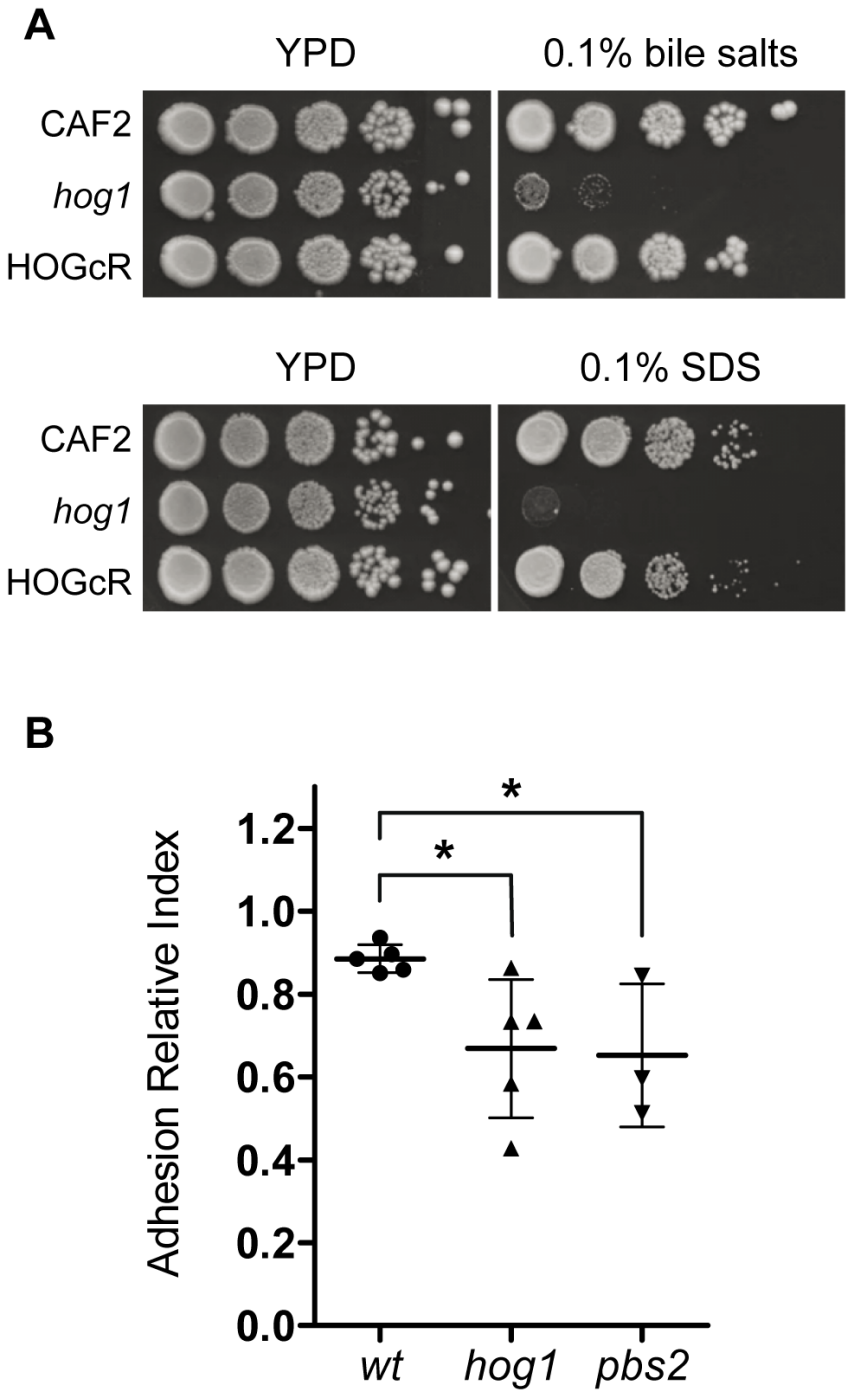
Mucosa adhesion and sensitivity to bile salts and SDS. A) Samples of 10-fold dilutions from stationary growing cells were spotted on YPD plates supplemented or not with 0.1% bile salts or 0.1% SDS (as indicated) and incubated at 37°C for 48 hours before being scanned. B) CAF2-dTOM2 was used as internal control to assess the capability to adhere gut mucosa of CAF2-GFP (wt, circles), *hog1*-GFP (*hog1*, triangles) and *pbs2*-GFP (*pbs2*, inverted triangles) strains. It is represented as each single independent value and mean ± standard deviation (from three to five different experiments) of the Adhesion Relative Index, that is calculated by dividing the adhered percentage of cells from (CAF2, *hog1* or *pbs2*)-GFP strains, recovered after 150 minutes of interaction with gut mucosa, by their percentage in the inoculum. *p<0.05.

We tried to determine the factors that could explain defects in colonization in the gastrointestinal tract. We tested *in vitro* the sensitivity of *hog1* to different ranges of pH and trypsin or pancreatic lipase activities, all of which may have a significant implication in gut colonization or passage through the intestinal tract. Although in these cases no differences were observed when comparing to the wt (data not shown), the *hog1* mutant was interestingly found to be sensitive to bile salts (Figures 6A and S4) being unable to grow at concentrations of 0.1%. A similar behaviour was seen for the ionic detergent SDS. Both phenotypes are also present in the *pbs2* mutant (data not shown), therefore suggesting that bile salts are a potential mechanism controlling fungal colonization that cannot be compensated in the absence of Hog1 activation.

We also tested the ability of *hog1* and *pbs2* mutants to adhere to the mouse gut. For this purpose we developed a competitive screening that can efficiently determine the behaviour of two differentially labeled strains. In our novel *ex vivo* adhesion assay, both *hog1* and *pbs2* cells showed a clear reduction in the adhesion to gut mucosa, with an Adhesion Relative Index of 0.67 (*hog1*) and 0.65 (*pbs2*) vs. 0.89 in CAF2 (p<0.05) after 150 minutes of interaction (Figure 6B). These results point out that *hog1* altered adhesion may contribute for its defective gut colonization.

## Discussion

Gastrointestinal models of colonization [14] are an excellent way to analyse *C. albicans*-host interactions. While these models are still defective in simulating certain aspects of human pathogenesis (such as too high fungal levels), they more closely mimic the normal route by which *C. albicans* accesses the bloodstream causing invasive diseases. They therefore provide an opportunity to analyse those factors influencing not only host tissue damage, but also those related to colonization and dissemination. Parallel with this development, it is necessary to improve methods to analyse the behaviour of fungal populations in the gut. For this main purpose, we have developed in this work an *ex vivo* flow cytometry-based methodology that is due to the complementary spectral properties of fluorescent proteins expressed in *C. albicans.* Both dTOM2 and GFP (used here) are stable enough to provide a strong signal, despite the reduced availability of oxygen within this particular niche, and allow differentiation of fungi from bacterial cells and other intestinal debris. Our data indicate that there is an overall good correlation between cytometry and standard CFUs counts, suggesting that -most likely- fungal gut content represent alive cells. An important technical feature of this method is that it may allow direct *ex vivo* analysis of specific parameters in fungal cells (in stools or gut content) without further manipulations (such as growth on liquid or solid cultures) that could hinder their physiological status. While bacterial content of human stools has been analysed by flow cytometry [56], several authors have revealed the existence of fungi in experimental mice gut just using microscopic analysis by staining the samples with calcofluor white [57] or specific antibodies [58], [59] or DNA probes [59]. This is, to our knowledge, the first demonstration that fungal population can be efficiently traced in stools *ex vivo* by flow cytometry. It therefore provides an opportunity to quantify parameters such as oxidative stress, metabolic status and cell wall architecture *in situ*, which can differ substantially from those obtained from *in vitro* cultures. In fact, a recent study describes how passage through the gastrointestinal tract triggers in *C. albicans* a developmental program that enables adaptation to the commensal state that significantly changes the morphology and transcriptional status of the cell [19].

Using this methodology -as well as the standard CFU counts-, we have further characterized the role that mice microbiota plays during *C. albicans* commensalism. In this model, where stable colonization levels of ≈10^7^ yeast cells/g of feces for several months is achieved without noticeable damage or pathogenesis to the host, reduction of intestinal microbiota is critical to allow *C. albicans* establishment in the gut [14], [15], [40]; however, previous studies did not reveal whether antibiotic therapy was required in already established *C. albicans* cells. We have found that, despite maintaining long-term high level colonization (10^6^–10^7^ CFU/g for up to 43 days), once antibiotic therapy is depleted there is a sharp drop in the detection of *C. albicans* in stools to levels of about 200 yeast cells/g after 14 days, indicating that mouse microbiota efficiently outcompetes with the already and long term-adapted *C. albicans* cells. This process is reversible and restoration of the antibiotic therapy allows high levels of fungus population in feces. While our data normally refers to analysis of viable cells in stools, that would not necessarily correlate with intestinal loads, but in our experience post mortem analysis of animals reveals a good correlation between both types of samples. Our data also reveal a failure to colonize mouse gut after establishment of the antibiotic therapy once CFUs counting drops behind our limit of detection (around 1.7×10^2^ CFU/g in stools), in accordance with the data obtained by Kinneberg at day three after orally inoculation of *C. albicans* [40]. Furthermore, addition of oral antibiotic treatment to those mice did not show any presence of *C. albicans*, while re-inoculation by gavage of 10^7^ cells at this moment does efficiently restore high fungal loads (data not shown). These observations strongly suggest that *C. albicans* cells do not actually persist in the mouse gut in a cryptic location and/or status when outcompeted by commensal microbiota. This is also consistent with the fact that this fungus is not a normal member of mice commensal microbiota while *C. tropicalis* is [60].

Having developed this model, we have determined the role that MAPK signaling may play in colonization. For this purpose, we established a competitive methodology in which two different labeled *C. albicans* strains are allowed to start colonization in the mouse gut. Expression of different fluorescent markers using the TET-OFF system does not significantly influence their *in vitro* or *in vivo* fitness in this particular niche, making them appropriate for *in vivo* analysis. However, this methodology does not allow screening a large number of mutants, as occurs with bar-coded strain collections [61], [62]. The latter strategy is useful for systemic screening of several mutants and has been shown valuable for the identification of virulence determinants [63] and transcription factors influencing commensalism [17]; however, simultaneous screening of large mutant collections require a parallel reduction in the starting number of individual clones, which could generate a “bottleneck” effect in these type of studies maybe influencing their outcome. Assessing individual clones via our fluorescent genetic labeling is therefore a valuable (and not as costly as qPCR) complementary tool. Such in deep studies have revealed the role that certain factors like *EFH1* play in the gastrointestinal tract while not having such role during a systemic infection [18]. A similar strategy has been used to analyse the role of the mating type locus (*MTL*) in virulence and competitiveness in a mouse systemic model by comparing either unlabeled or GFP-labeled strains that were homozygous or heterozygous for *MTL* [64].

We have addressed here the role that three MAPK pathways play in mice gut colonization. Both the SVG and cell integrity pathway seem to have a similar role in gut establishment by *C. albicans* since both MAPK mutants (*cek1* and *mkc1*) strains show a similar phenotype. These two signaling routes have been related to the response to cell wall damage [25], [26], [34], [65], [66], suggesting that this phenotype could be responsible for this behaviour. However, we have focused on the HOG pathway as this one is critical for colonization of mice, since mutants in this pathway are unable to establish or maintain high fungal burdens. We have demonstrated this not only by the use of mutants defective in the pathway (*hog1* and *pbs2*) but also by the development of a genetic strategy where *in vivo* (in gut) deletion of the analysed gene is accomplished, a strategy similar to the FLP-mediated excision of the *URA3* marker that allows measuring transcriptional activity in the gut [19]. The scarce detection of mutant *hog1* cells during competition in colonization, only in the first few days, indicates transient passage through the gastrointestinal tract but not true colonization. It must be emphasized that while *hog1* mutants are unable to establish in the gut in the presence of similar amounts of wild type cells, they can do it in its absence reaching high fungal loads for about 10 days. This could suggest the existence of either defined and limited niches within the gut where *C. albicans* wild type proliferation can take place (competing and therefore, displacing *hog1* mutant cells) or a clear fitness defect for *hog1* mutants *in vivo*. At the moment, we can only speculate about the reasons for this behaviour. Oxidative and osmolarity stress are main mechanisms for the activation of the HOG pathway and *hog1* strain is sensitive to both oxidative and osmotic stress [30]. It has been shown that a highly anaerobic environment can lead to transient ROS production [67] and, moreover, *hog1* cells are more efficiently killed by promyelocytic cells and macrophages *in vitro* [52], suggesting that mucosal phagocytes may be an important factor controlling establishment of the fungus. Alternatively, osmolarity could be important within certain locations of the lumen although this remains to be determined experimentally. Another important feature revealed in this work is the sensitivity to bile salts and SDS, a novel phenotypic trait characteristic of *hog1* mutants that could contribute to its altered colonization. However, we have seen that a *hog1* mutant is able to survive in the gut for few days when inoculated alone and also that *hog1*-phenotype cells from HOGcR strain are found in similar proportion in all gut locations analysed. This suggests that there is no correlation with bile salts concentration in the gut, arguing against this factor being the main mechanism determining colonization. Besides, it has been seen, by microscopic examination of mouse gut tissues, that a layer of fungus exists embedded in adherent bacteria [59]. In this work, by using a competitive assay we describe that *hog1* cells adhere less efficiently to the mouse gut mucosa, with a relative index of 0.67 versus 0.89 for wild type cells; these effects are apparently mild, but could be crucial *in vivo* under conditions in which the organism would have a physical or temporal window for colonization competing with endogenous microbiota. It has been described that *hog1* mutants display an altered sensitivity to cell wall interfering drugs [29] and how the HOG pathway regulates to a certain extend the biogenesis of the cell wall [34], [35]. Moreover, proteomic and transcriptomal analyses also revealed variations in cell wall proteins [68], [69], therefore providing a reasonable explanation for the altered adhesion to host surfaces. Filamentation is enhanced in *hog1* mutants under defined conditions [29] which can also alter adhesion to certain surfaces. Therefore, reduced colonization can result from a complete transcriptional program present in HOG competent cells.

Additionally, a different host immune response against *hog1* mutants may happen. It is interesting to note that, after *hog1* levels have notably decreased, boosting with the same strain (*hog1*-GFP) did not follow the same pattern of colonization: it did not reach previous high sustained CFU levels and the kinetic of loss was much more pronounced (reduction of 1.24 versus 0.324 logarithmic units per day). We have also observed a slight impairment in CAF2-RFP strain in initiating colonization after inoculation of mice previously colonized with *hog1*-GFP strain. Those observations would suggest an induction of host adaptive defenses against *C. albicans* colonization.

While further work is needed to better understand changes occurring in *C. albicans* during *in vivo* colonization, the ability to use specific *C. albicans* mutants defective in colonization but with a transient passage through gut may lead to interesting approaches in fields of vaccine and immunotherapy to fungal infections.

## Supporting Information

Figure S1 **Alignment of dTomato and** ***C. albicans*** **adapted dTOM and dTOM2.** An alignment between Shaner original dTomato [47] and versions adapted to *C. albicans* dTOM [70] and dTOM2 (this work) is shown. Alignment was generated using the ClustalW server at EBI (http://www.ebi.ac.uk/Tools/msa/clustalw2/). Numbers indicate nucleotide position number relative to the own origin. Parameters were left to their default values. (*) indicates a residue conserved in all sequences, (:) indicates conservation between groups of strongly similar properties, (.) indicates conservation between groups of weakly similar properties.
(TIF)

Figure S2 **Growth behaviour of** ***C. albicans*** **expressing either GFP or dTOM2** ***in vitro*** **and** ***in vivo*** **.** A) Correlation between CFUs and FACS particle quantification. Samples (n = 209) from several colonization assays were analysed by both CFU counting and FACS particle quantification for gated fluorescent populations. Logarithmic values obtained from each sample were plotted and compared. B) Growth curve of CAF2 and MAPK mutant strains expressing GFP or dTOM2. Cells were diluted at O.D._600_ = 0.1 from a stationary phase culture in YPD medium supplemented or not with 20 µg/mL doxycycline. C) Either a pure culture of CAF2-dTOM2 or a 1 1 population of CAF2-dTOM2/CAF2-GFP were inoculated in SD medium at O.D._600_ = 0.1 and allowed to grow until stationary phase at 37°C. Periodically (2–3 days), the culture was diluted again to the same initial O.D. and the percentage of dTOM2 expressing cells (red colonies) was estimated out of the total number of CFUs. D) Competition colonization assay with different fluorescent proteins. CFUs are represented for each individual as red circles (CAF2-dTOM2) or green circles (CAF2-GFP). Colored lines reflect the tendency of the median of the respective strain. Oral antibiotic therapy (streptomycin, bacitracin and gentamycin) was given to mice (n = 3) from 4 days before the gavage of 10^7^ cells of a 1 1 mixture of CAF2-dTOM2 and CAF2-GFP. CFUs were counted and associated with each strain based on their pattern of FP expression on solid SD plates.
(TIF)

Figure S3 **Controls and strategy for** ***in vivo*** **gene excision.** A) *C. albicans* TET-ON expression during colonization assay. *C. albicans* is represented for each individual as open circles (population determined by CFU) or green circles (population expressing GFP determined by FACS quantification). Lines reflect the tendency of the median of the respective population. Oral antibiotic therapy (streptomycin, bacitracin and gentamicin) was given to mice (n = 2) from 4 days before a gavage of 10^7^ cells of CAF2-GFPind strain (day 0). Autoclaved chlortetracycline (1 mg/mL aCT) was added to the standard antibiotic treatment to induce the expression of GFP gene (yellow arrow). B) Schematic representation of the genetic strategy to obtain a tetracycline-dependent mutant for *HOG1*. C) *in vitro* excision of *HOG1* ectopic gene in the HOGcR strain. Cells were periodically diluted from a stationary phase culture in YPD medium at O.D._600_ = 0.1 and 25 µg/mL aCT was added to induce the expression of FLP gene (yellow arrow). CFUs from each population are represented as closed diamonds (wt phenotype) or open triangles (*hog1* phenotype). Percentage of osmo-tolerant CFUs was determined based on their pattern of osmo-sensitivity on 1.5 M sorbitol solid YPD plates.
(TIF)

Figure S4 **Sensitivity to bile salts and SDS of MAPK mutants in** ***C. albicans*** **.** Samples of 10-fold dilutions from stationary growing cells were spotted on YPD plates supplemented with 0.1% bile salts or SDS (as indicated) and incubated at 37°C for 24 hours before being scanned.
(TIF)

Table S1 ***C. albicans*** **strains used in this work.** All *C. albicans* strains used derive from the SC5314 clinical isolated. Genotype and reference from each strain are indicated. A nomenclature has been established for easily follow the text and the figures.
(DOC)
