# Supplementary figures and images for: The HOG Pathway Is Critical for the Colonization of the Mouse Gastrointestinal Tract by Candida albicans
Source: PLoS One. 2014 Jan 27;9(1):e87128. doi: 10.1371/journal.pone.0087128 (PMC3903619; doi:10.1371/journal.pone.0087128)

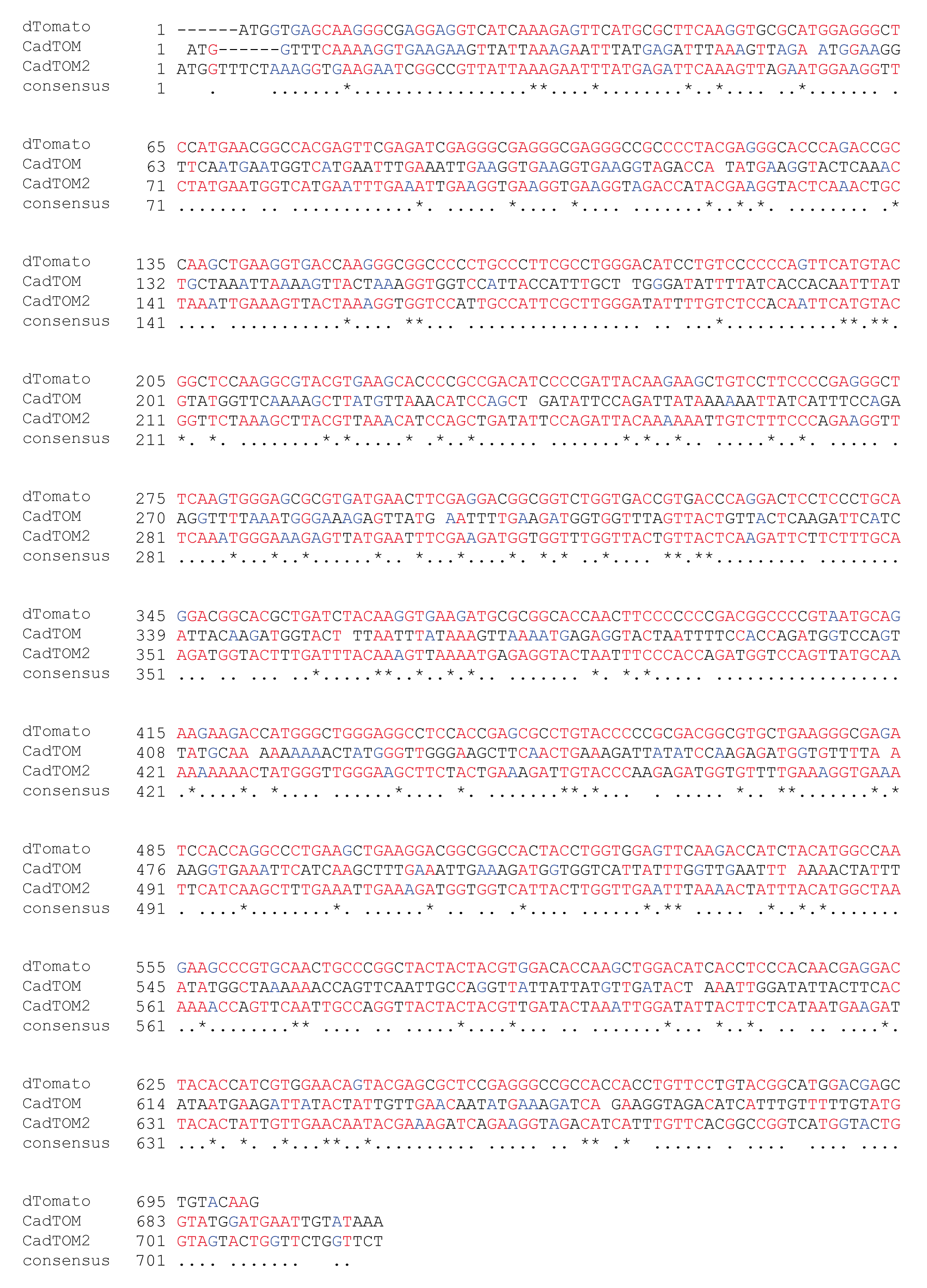

Supplement: Figure S1 — Alignment of dTomato and C. albicans adapted dTOM and dTOM2. An alignment between Shaner original dTomato [47] and versions adapted to C. albicans dTOM [70] and dTOM2 (this work) is shown. Alignment was generated using the ClustalW server at EBI (http://www.ebi.ac.uk/Tools/msa/clustalw2/). Numbers indicate nucleotide position number relative to the own origin. Parameters were left to their default values. (*) indicates a residue conserved in all sequences, (:) indicates conservation between groups of strongly similar properties, (.) indicates conservation between groups of weakly similar properties. (TIF) [file pone.0087128.s001.tif]

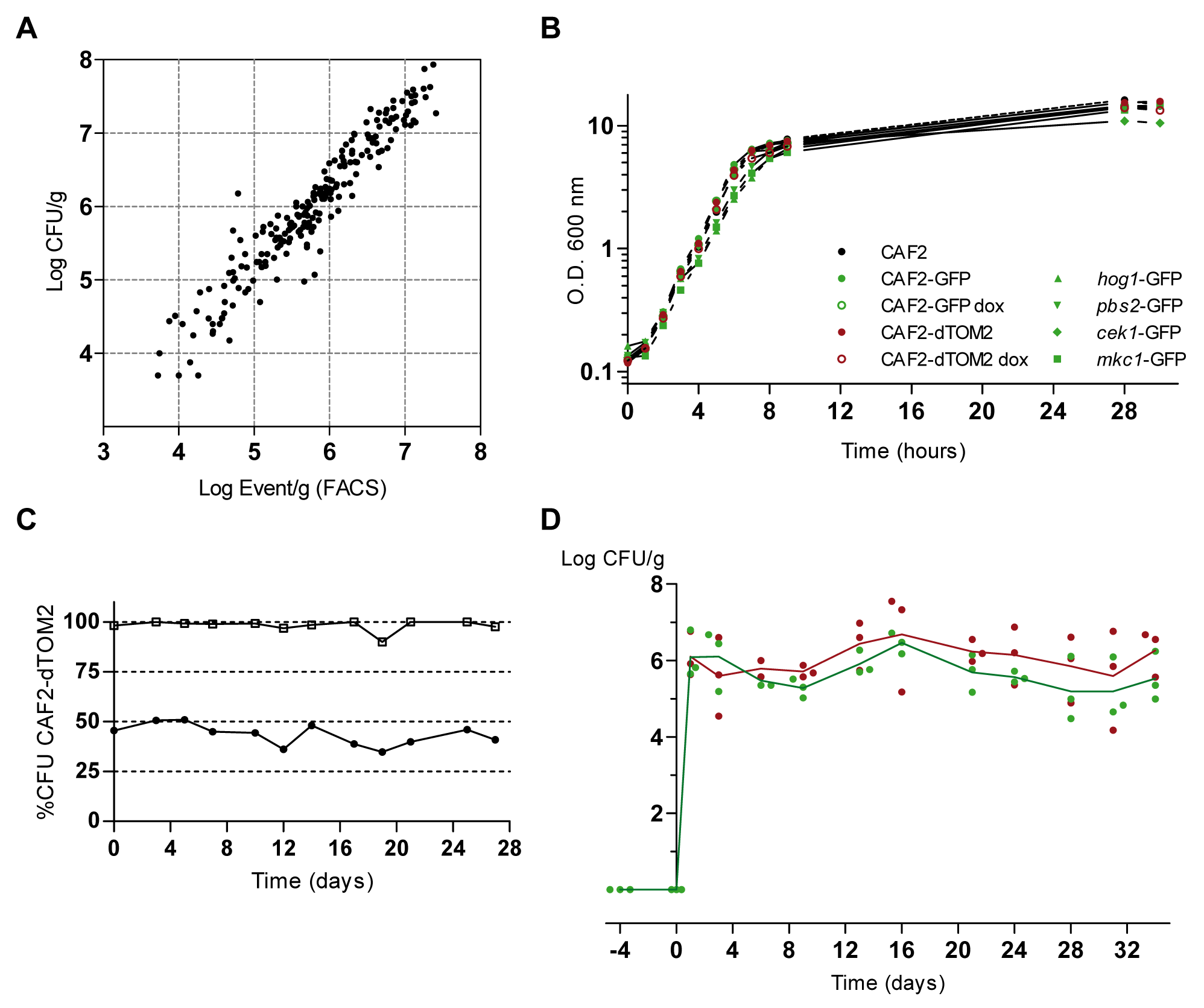

Supplement: Figure S2 — Growth behaviour of C. albicans expressing either GFP or dTOM2 in vitro and in vivo . A) Correlation between CFUs and FACS particle quantification. Samples (n = 209) from several colonization assays were analysed by both CFU counting and FACS particle quantification for gated fluorescent populations. Logarithmic values obtained from each sample were plotted and compared. B) Growth curve of CAF2 and MAPK mutant strains expressing GFP or dTOM2. Cells were diluted at O.D.600 = 0.1 from a stationary phase culture in YPD medium supplemented or not with 20 µg/mL doxycycline. C) Either a pure culture of CAF2-dTOM2 or a 1 1 population of CAF2-dTOM2/CAF2-GFP were inoculated in SD medium at O.D.600 = 0.1 and allowed to grow until stationary phase at 37°C. Periodically (2–3 days), the culture was diluted again to the same initial O.D. and the percentage of dTOM2 expressing cells (red colonies) was estimated out of the total number of CFUs. D) Competition colonization assay with different fluorescent proteins. CFUs are represented for each individual as red circles (CAF2-dTOM2) or green circles (CAF2-GFP). Colored lines reflect the tendency of the median of the respective strain. Oral antibiotic therapy (streptomycin, bacitracin and gentamycin) was given to mice (n = 3) from 4 days before the gavage of 107 cells of a 1 1 mixture of CAF2-dTOM2 and CAF2-GFP. CFUs were counted and associated with each strain based on their pattern of FP expression on solid SD plates. (TIF) [file pone.0087128.s002.tif]

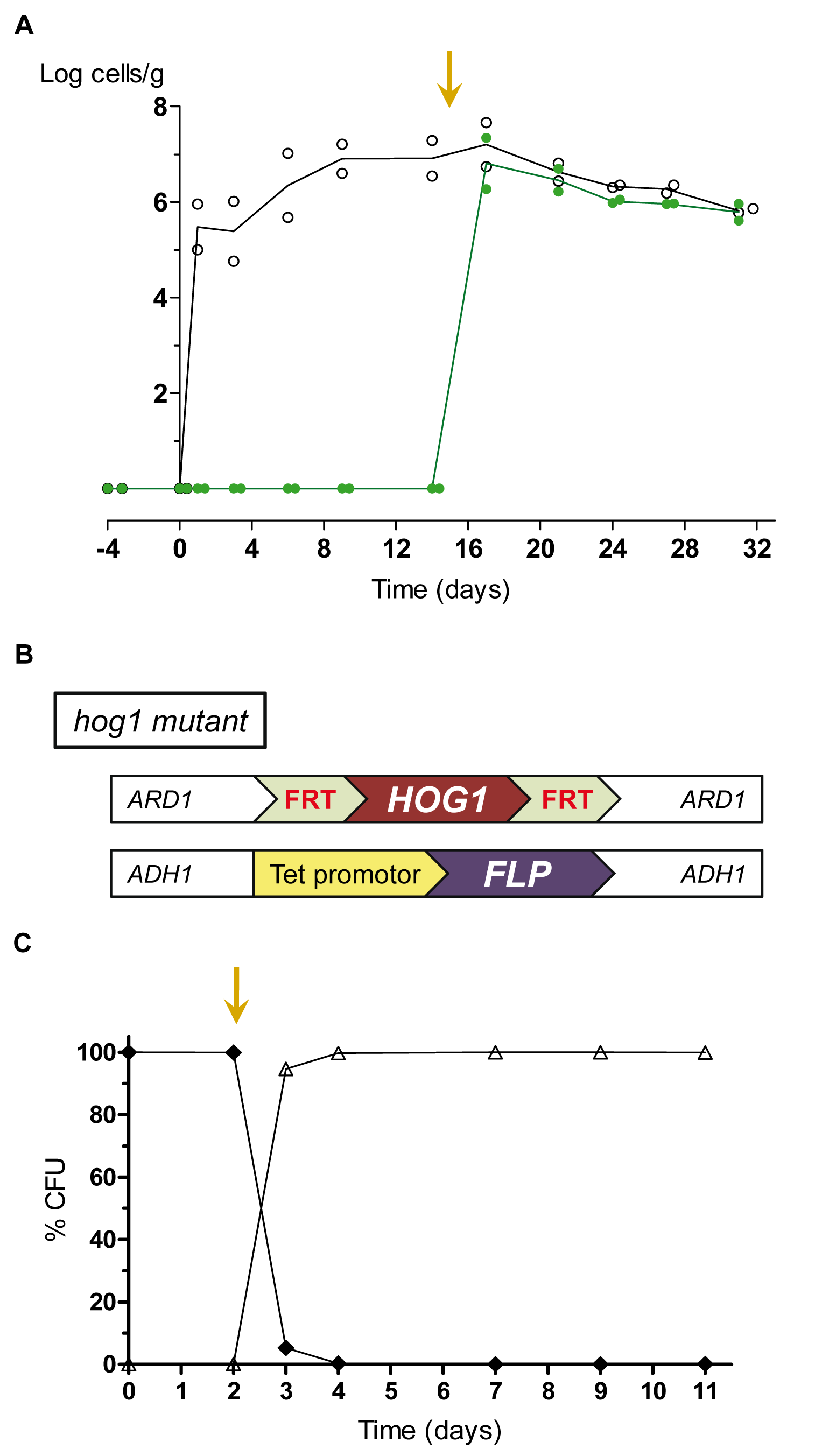

Supplement: Figure S3 — Controls and strategy for in vivo gene excision. A) C. albicans TET-ON expression during colonization assay. C. albicans is represented for each individual as open circles (population determined by CFU) or green circles (population expressing GFP determined by FACS quantification). Lines reflect the tendency of the median of the respective population. Oral antibiotic therapy (streptomycin, bacitracin and gentamicin) was given to mice (n = 2) from 4 days before a gavage of 107 cells of CAF2-GFPind strain (day 0). Autoclaved chlortetracycline (1 mg/mL aCT) was added to the standard antibiotic treatment to induce the expression of GFP gene (yellow arrow). B) Schematic representation of the genetic strategy to obtain a tetracycline-dependent mutant for HOG1. C) in vitro excision of HOG1 ectopic gene in the HOGcR strain. Cells were periodically diluted from a stationary phase culture in YPD medium at O.D.600 = 0.1 and 25 µg/mL aCT was added to induce the expression of FLP gene (yellow arrow). CFUs from each population are represented as closed diamonds (wt phenotype) or open triangles (hog1 phenotype). Percentage of osmo-tolerant CFUs was determined based on their pattern of osmo-sensitivity on 1.5 M sorbitol solid YPD plates. (TIF) [file pone.0087128.s003.tif]

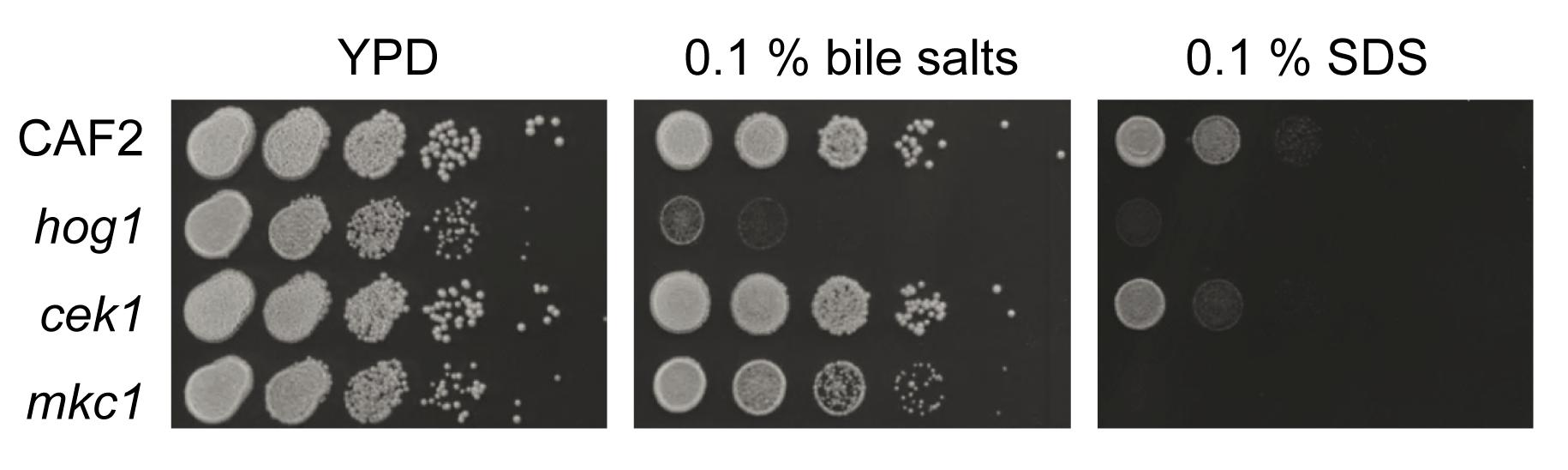

Supplement: Figure S4 — Sensitivity to bile salts and SDS of MAPK mutants in C. albicans . Samples of 10-fold dilutions from stationary growing cells were spotted on YPD plates supplemented with 0.1% bile salts or SDS (as indicated) and incubated at 37°C for 24 hours before being scanned. (TIF) [file pone.0087128.s004.tif]
