# Supplementary material for: The HOG Pathway Is Critical for the Colonization of the Mouse Gastrointestinal Tract by Candida albicans
Source: PLoS One. 2014 Jan 27;9(1):e87128. doi: 10.1371/journal.pone.0087128 (PMC3903619; doi:10.1371/journal.pone.0087128)
Supplement: Table S1 — C. albicans strains used in this work. All C. albicans strains used derive from the SC5314 clinical isolated. Genotype and reference from each strain are indicated. A nomenclature has been established for easily follow the text and the figures. (DOC) [file pone.0087128.s005.doc]

### Table S1. *C. albicans* strains used in this work.

| **Strain** | **Genotype** | **Nomenclature** | **Source** |
| --- | --- | --- | --- |
| SC5314 |  |  | [41] |
| CAI4 | *ura3Δ::imm434/ura3Δ::imm434* |  | [42] |
| RM1000 | CAI4 *his1::hisG/his1::hisG* |  | [50] |
| CAF2 | *ura3Δ::imm434/URA3* | wt | [42] |
| BRD3-37 | RM1000 *pbs2Δ::cat/pbs2Δ::cat-URA3-cat* | *pbs2* | [44] |
| CK43B-16 | CAI4 *cek1Δ::hisG/cek1Δ::hisG-URA3-hisG* | *cek1* | [43] |
| CM1613 | CAI4 *mkc1Δ::hisG/mkc1Δ::hisG-URA3-hisG* | *mkc1* | [25] |
| HI3-21 | CAI4 *hog1::hisG/hog1::hisG-URA3-hisG* | *hog1* | This study |
| HI7-6 | CAI4 *hog1::hisG/hog1::hisG* | *hog1ura3* | This study |
| COA6-3 | CAF2 *ADH1/adh1::tTA P_tet_ -GFP-SAT1* | CAF2-GFP | This study |
| PAP1 | CAF2 *ADH1/adh1::rtTA P_tet_ -GFP-SAT1* | CAF2-GFPind | This study |
| PPD7 | CAF2 *ADH1/adh1::tTA P_tet_ -dTOM2-SAT1* | CAF2-dTOM2 | This study |
| COA10-9 | *hog1 ADH1/adh1::tTA P_tet_ -GFP-SAT1* | *hog1*-GFP | This study |
| PPD15 | *pbs2 ADH1/adh1::tTA P_tet_ -GFP-SAT1* | *pbs2*-GFP | This study |
| PPD11 | *cek1 ADH1/adh1::tTA P_tet_ -GFP-SAT1* | *cek1*-GFP | This study |
| PPD16 | *mkc1 ADH1/adh1::tTA* *P_tet_-GFP-SAT1* | *mkc1*-GFP | This study |
| PPD20 | *hog1ura3* *ARD1/ard1::FRT-HOG1-SAT1-FRT* | HOGfR | This study |
| PPD21 | *hog1ura3* *ARD1/ard1::FRT-HOG1-SAT1-FRT ADH1/adh1::rtTA P_tet_-FLP-URA3* | HOGcR | This study |
